# Stem Cell Therapy: a Novel Approach for Vision Restoration in Retinitis Pigmentosa

**Published:** 2013

**Authors:** Harvey Siy Uy, Pik Sha Chan, Franz Marie Cruz

**Affiliations:** 1 Department of Ophthalmology and Visual Sciences, University of the Philippines, Philippine General Hospital, Manila,; 2 St. Luke's Medical Center, Quezon City,; 3 Pacific Eye and Laser Institute, Makati City, Philippines

**Keywords:** Pluripotent stem cells, Regenerative medicine, Retinitis pigmentosa, Stem cell therapy

## Abstract

Unfortunately, at present, degenerative retinal diseases such as retinitis pigmentosa remains untreatable. Patients with these conditions suffer progressive visual decline resulting from continuing loss of photoreceptor cells and outer nuclear layers. However, stem cell therapy is a promising approach to restore visual function in eyes with degenerative retinal diseases such as retinitis pigmentosa. Animal studies have established that pluripotent stem cells when placed in the mouse retinitis pigmentosa models have the potential not only to survive, but also to differentiate, organize into and function as photoreceptor cells. Furthermore, there is early evidence that these transplanted cells provide improved visual function. These groundbreaking studies provide proof of concept that stem cell therapy is a viable method of visual rehabilitation among eyes with retinitis pigmentosa. Further studies are required to optimize these techniques in human application. This review focuses on stem cell therapy as a new approach for vision restitution in retinitis pigmentosa.

## INTRODUCTION

At present, degenerative retinal diseases such as retinitis pigmentosa (RP) and dry type age related macular degeneration (AMD) remain untreatable. Patients with these conditions suffer progressive visual decline resulting from continuing loss of photoreceptor cells and outer nuclear layers. Both disorders are characterized by the progressive dysfunction and death of the light sensing photoreceptors of the retina and other supportive cells. Because of the limited regenerative capacity of the mammalian retina, retinal progenitor cell are being developed to serve as “spare parts” for lost photoreceptor cells. Advances in molecular biology have identified innovative approaches that, for the first time, provide hope for permanent visual rehabilitation. Stem cell therapy can potentially replace lost photoreceptor and retinal pigment epithelial cell and subsequently restore visual function in eyes with degenerative retinal disorders [1].

## HYPOTHESIS

**Figure 1:**
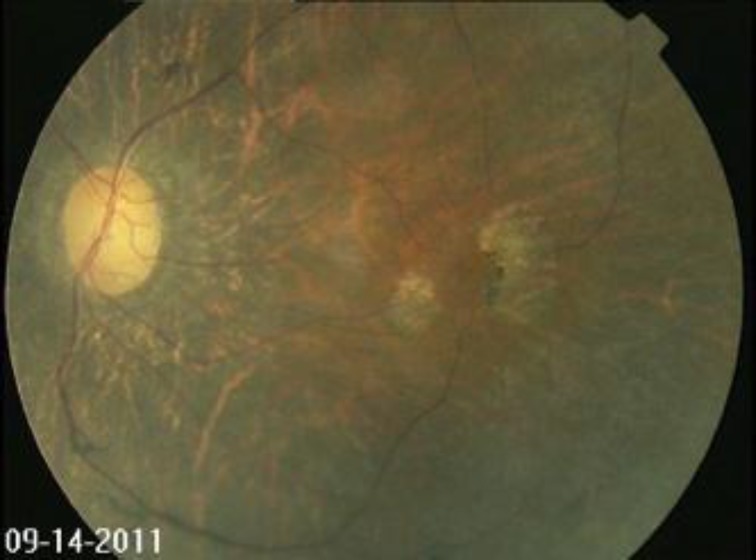
Fundus photograph of eye with advanced retinitis pigmentosa. Note encroachment of the pigmentation into the macula, patchy loss of retinal pigment epithelial layers, attenuation of retinal blood vessels and optic nerve pallor

A potential target disease for stem cell therapy is retinitis pigmentosa (RP). RP is the most commonly inheritable eye disease that causes progressive loss of photoreceptor cells resulting in gradual visual decline. While the onset of RP may occur during infancy, the first symptoms are usually observed in early adulthood, beginning with nyctalopia or night blindness followed by loss of peripheral vision and eventually, as the central photoreceptors in the macula are damaged, loss of fine central vision. Morphologically, these retinas are characterized by centripetal proliferation of bone spicule-like pigmentation, attenuation of retinal blood vessels and optic nerve pallor (Figure 1). At least 50 genetic mutations have been associated with the disease. The Beijing Eye Study reported a prevalence rate of 1 in 1000 and estimates about 1.3 million people are afflicted in China alone [2].

Transplantation of progenitor stem cells that can be stimulated to become replacement photoreceptors and supportive outer retina cells can theoretically lead to treatments that restore visual function [3]. Recently, the U.S. Food and Drug Administration approved phase I/II clinical trials for stem cell-based retinal pigmented epithelium (RPE) transplantation. Several issues surrounding stem cell use need to be addressed. When is stem cell therapy indicated? What type of stem cells to use and at what dosage? How to safely implant into the target tissues? How to efficiently stimulate stem cells into the desired development pathway? What are the side effects? What is the duration of effect? This article will review some of the developments in this field of regenerative medicine for the treatment of degenerative retinal diseases. 

## DISCUSSION

Two types of stem cells may be utilized to produce retinal progenitor cells. Firstly, Embryonic stem (ESC) and secondly, induced pluripotent stem (IPS) cells. Both types of cells are pluripotent and capable of becoming any cell type. ESC’s are derived from embryos while IPS cells are obtained from a variety of adult tissues such as skin, bone marrow, teeth. IPS cells, if successfully implanted and optimized, can potentially provide an unlimited supply of stem cells for transplantation. 

Cell replacement is one approach for restoration of vision in RP. Because visual loss usually occurs when the outer retinal photoreceptor layer is lost, therapeutic timing should be at this stage of disease. Singh and colleagues have demonstrated using a murine model of severe human retinitis pigmentosa, that at a stage when no host rod cells are remaining, transplanted rod precursors can reestablish an anatomically distinct and appropriately polarized outer nuclear layer. In their study, restoration of a trilaminar retinal organization was restored to RD1 hosts with only two retinal layers before treatment. The introduced rod precursors continued to develop in the host niche to become mature rods complete with light-sensitive outer segments and connections to host neurons downstream. Visual function was also restored. These findings indicated that stem cell therapy may reinstate a light-sensitive cell layer de novo and restore structurally damaged visual circuits. In this model, total photoreceptor layer reconstruction is one approach to further develop cell-based strategies for retinal repair [4].

ESC’s have been shown to generate functional photoreceptor cells restoring light response of photoreceptor-deficient mice, but there is concern over the risk for tumor formation using ESC. Li and colleagues successfully cultured Nestin(+)Sox2(+)Pax6(+) multipotent retinal stem cells (RSCs) from the adult mouse retina. These ESC’s are capable of producing functional photoreceptor cells that restore light response of photoreceptor-deficient RD1 mutant mice. After several cycles of expansion using growth factors, cultured RSCs still maintained proliferation and differentiation potential [5].

Under optimized differentiation conditions, ESC’s can differentiate into all the major retinal cell types found in the adult retina such as photoreceptor cells under optimized differentiation conditions. Following transplantation into the subretinal space of slowly degenerating RD7 mutant eyes, RSC-derived photoreceptor cells were shown to integrate into the retina, and develop into cells morphologically resembling endogenous photoreceptors and forming synapses with resident retinal neurons. When transplanted into eyes of photoreceptor-deficient RD1 mutant mice, an RP model, RSC-derived photoreceptors can partially restore light response, indicating functionality. In animal studies, no evidence for tumor development was found [5]. 

Along similar lines, autologous IPS cells are being developed for stem cell transplantation. This lack of immunogenicity confers an important advantage. Because IPS cells are autologous or derived from the same organism, they do not incite immunological reaction nor require use of immunosuppressive medication [6]. 

Li and colleagues recently transplanted human IPS cell-derived retinal pigment epithelium (RPE) cells into the subretinal spaces of mouse models with the Rpe65rd12 /Rpe65rd12 form of RP. A healthy adult provided skin fibroblasts cultured with lentivirus-delivered genes encoding transcription factors OCT4, SOX2, KLF4, and MYC. Antibody staining of markers (TRA-1-60, SSEA4, NANOG, and SOX2) and a teratoma assay demonstrated pluripotency of the hiPS cells. Culturing in differentiation medium guided their fate to RPE. By 12 weeks, from 30% to 50% of the surfaces of 12-well dishes were coated with RPE with characteristic hexagonal shapes, perinuclear melanin granules, and microvilli [6]. 

The target mice had albinism, which provided a white contrast against which the transplanted pigmented cells would be visible. The mice also had severe combined immune deficiency to prevent graft-vs-host disease. An injection of 1000 hiPS-derived RPE cells was administered into the subretinal space in the right eyes of 34 mice at two days following birth. The mice were sacrificed at six months, shortly before they would have died from severe combined immune deficiency [6].

Successful development into RPE cells was indicated by: 1) microscopic confirmation of pigmented hiPS-derived RPE admixed into the native, albino RPE; 2) quantitative polymerase chain reaction detection of markers of human fetal RPE and IPS-derived RPE; 3) positive staining for rhodopsin indicating that the hiPS-derived RPE cells phagocytosed photoreceptors. Furthermore, in some mice, electroretinogram (ERG) response to measure neuronal function, demonstrated a small but significant improvement of mean β-wave peak difference between treated and control eyes of 13.7 μV (P = 0.0246). Furthermore, no tumor growth was observed [6]. 

The use of retinal progenitor sheet transplantation is another promising approach. Seller and Aramant demonstrated that when freshly dissected sheets of fetal-derived retinal progenitor cells are mixed with RPE and transplanted subretinally, improvements of visual acuity are observed among animals and humans. Visual improvement in this model is attributed to restoration of synaptic connections between transplant and host when transplant processes proliferate into the inner plexiform layer of the host retina and presumably form synapses. One drawback of widespread use of this method is limited supply of fetal donor tissue [7].

Future areas for stem cell development include methods for optimizing stem cell production and delivery. The use of specific extracellular matrix can stimulate the development of human pluripotent stem cells into transient organized neuroepithelum with rapid differentiation into retinal progenitor cells [8]. Garit-Hernandez and colleagues reported that by replicating the hypoxic stages of retinal development, an increase in number of retinal cells (Crx-positive, S-Opsin-positive, rhodopsin/recoverin positive cells) derived from ESC transplanted in the subretinal space of wild type mice [9]. Some promising work has been reported on increasing the yield of differentiated rod photoreceptor genes by using the conjunctiva mesenchymal stem cells on nanofibrous scaffolds [10].

## CONCLUSION

Previously, RP was considered a devastating and untreatable condition. These pioneering animal studies provide hopeful evidence for the hypothesis that stem cell therapy is a viable means for visual rehabilitation of RP patients. What is now known is that stem cell therapy can potentially replace degenerate photoreceptors and outer retinal cells. When placed in the appropriate tissue niche, these stem cells not only survive but differentiate into critical retinal cells, develop a retina-like organizational structure and exhibit functional characteristics of full-fledged photoreceptors and outer retinal cells. Further studies are needed to optimize techniques and validate these findings before proceeding to human trials. 

## DISCLOSURE

Conflicts of Interest: None declared.
